# The Role of Anesthetic Drugs and Statins in Prostate Cancer Recurrence: Starting at the Actual Knowledge and Walking through a New Paradigm

**DOI:** 10.3390/cancers15113059

**Published:** 2023-06-05

**Authors:** Aida Raigon Ponferrada, Juan Carlos Molina Ruiz, Salvador Romero Molina, Verónica Rodriguez Garcia, Jose Luis Guerrero Orriach

**Affiliations:** 1 Institute of Biomedical Research in Malaga [IBIMA], 29010 Malaga, Spain; 2 Department of Anaesthesiology, Virgen de la Victoria University Hospital, 29010 Malaga, Spain; 3 Department of Pharmacology and Pediatrics, School of Medicine, University of Malaga, 29010 Malaga, Spain; 4 Hospital Virgen de la Victoria, Campus Teatinos CP Malaga, 29010 Malaga, Spain

**Keywords:** surgery and inflammatory response, anesthesia, analgesia, cancer recurrence, metastasis, anesthetics, opioids, statins, prostate cancer

## Abstract

**Simple Summary:**

The relationship between an anesthetic procedure and cancer is a common theme of various research papers and systematic reviews. Prostate cancer, although it has a high incidence, is one example for which we currently do not know the possible benefits of anesthetic techniques. Our article starts from the current knowledge of the influence of anesthetic drugs and anesthetic techniques, and we propose where we should go in the future to elucidate its role in prostate cancer recurrence.

**Abstract:**

Prostate cancer has become a major health problem in men. Its incidence is increasing as the average age of the affected population tends to be higher. Of all the possible treatments, surgery is the gold standard in its treatment. Surgery produces a deregulation in the immune system that can favour the development of distant metastases. Different anesthetic techniques have raised the hypothesis that different anesthetic drugs influence tumor recurrence and prognosis. Some mechanisms are beginning to be understood by which halogenated agents in cancer patients and the use of opioids may negatively affect patients. In this document, we group together all the available evidence on how the different anesthetic drugs affect tumor recurrence in prostate cancer.

## 1. Introduction

Cancer remains one of the diseases with the highest mortality and morbidity in the world. In cancer patients, the main cause of death is not the primary tumor, but tumor recurrence.

Prostate cancer has the highest incidence in men with tumor diseases of the urinary tract and is one of the main health problems of any health system.

**Figure 1:**
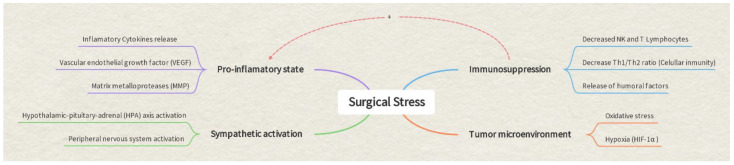
Surgical stress, inflammation and tumor relationship.

Many patients diagnosed with any type of tumor will require surgical intervention as part of their cancer treatment. However, surgery, with undoubted curative power, has also been related to the progression of tumor disease. Surgical manipulation results in a significant systemic release of tumor cells. Whether these cells cause metastasis depends largely on the balance between the aggressiveness of the tumor and the adaptability of the organism [1]. Different works have shown that surgical stress favors tumor recurrence by activating a sympathetic response, generating immunosuppression and deregulation of the inflammatory response. Postoperative immunosuppression can last for about 2 weeks and peaks 3 days after surgery [2]. The so-called “tumor microenvironment (TME)” determines the potential for tumor metastasis. Surgical intervention and tissue trauma easily disrupt the TME and promote the spread of residual cancer cells [3,4]. Surgical stress activates the hypothalamic–pituitary–adrenal (HPA) axis and the peripheral nervous system (PNS) through the release of endogenous prostaglandins and catecholamines, suppresses the activity of NK and Tc cells, which are the main immunosurveillance cells, and suppresses cell-mediated immunity [5,6,7]. Unfortunately, an imbalance between proinflammatory and anti-inflammatory responses can lead to the dysregulation of cellular immunity and subsequent immunosuppression [8,9,10,11,12,13,14]. (Figure 1).

The administration of anesthetic and analgesic drugs can promote or inhibit tumor spread by causing transient immune impairment at the time of greatest risk of oncological cell spread, such as during surgery.

Currently, prostate cancer is the most common malignancy among men, surpassed only by skin cancer. Prostate cancer is also the second leading cause of cancer death in men in the United States. In 2023, an estimated 288,300 men in the United States will be diagnosed with prostate cancer, accounting for 27% of all male cancers. It is the fourth most commonly diagnosed cancer in the world. Prostate cancer is also the second leading cause of cancer death in men in the United States [15].

The main treatment for prostate cancer is surgery. One of the main concerns among cancer patients is recurrence after cancer surgery. The results after radical retropubic prostatectomy depend on the local evolution of the tumor that can be defined by the risk in the D’Amico classification, the stage of lymph node involvement and the margins of the resected tumor [16]. In addition, certain factors in the perioperative period, such as the surgical manipulation of the tumor, need for blood transfusion, appearance of severe hypothermia, pain and surgical stress, can influence the risk of oncological recurrence [17]. Other factors possibly involved in tumor recurrence are anesthetics, the anesthetic technique, the use of analgesics to control acute pain and the administration of opioids [18,19].

The hypothesis that anesthetic drugs and techniques play a role in the recurrence of prostate cancer is based on other studies that have shown their association with the recurrence of other urological tumors, such as bladder tumors [20,21], since there is an important shortfall in evidence in prostate cancer. Retrospective studies on urinary tract tumors have compared overall survival with different anesthetic techniques without identifying either specific receptors or the pathways by which they modulate the cell cycle and produce such effects [20]. There is therefore a gap in understanding how and what influence on tumor recurrence, specifically in prostate cancer, the drugs that are commonly used in clinical practice have in the perioperative period. Our review aims to generate a unique document that collects the current evidence on the influence of drugs used in the perioperative period and their influence on recurrence in prostate cancer, in turn clarifying the molecular mechanisms from which they exert that action. We intend to generate a work that serves as a starting point for new studies that can relate the drugs and modulation pathways to which we refer to modulation in gene expression and that finally generate a specific indication for each anesthetic technique.

In the rest of this paper, we describe the following points:

2. Anesthesia and Prostate Cancer

2.1. Hypnotic Agents: Propofol and Halogenated Agents

2.2 Opioids

2.3 Regional Anesthesia

3. Statins

4. Discussion: Towards a New Paradigm

5. Conclusions

## 2. Anesthesia and Prostate Cancer

### 2.1. Hypnotic Agents: Propofol and Halogenated Agents

Propofol seems to have a protective effect against the tumor progression of prostate cancer, while inhaled agents have been described as pro-oncogenic, in line with other tumor lines such as breast [22] or colorectal cells [23].

Volatile anesthetics and intravenous propofol have distinct influences on both cancer cell biology and host immunity. Several in vitro studies have shown that the exposure of tumor cells to volatile anesthetics is associated with the increased expression of prometastatic and protumorigenic factors through signaling pathways including hypoxia-inducible factor 1α and transforming growth factor β/Smad [24], and cytoplasmic HIF-2α and nuclear p38, both related to a worse prognosis in cancer patients [25]. Sevoflurane has been shown to increase the levels of protumor cytokines and MMPs [26].

Propofol exerts a protective effect through various mechanisms, including an anti-inflammatory effect, COX-2 inhibition and PGE-2 reduction, increased CTL activity and decreased pro-inflammatory cytokines [27]. Propofol does not affect the Th1/Th2 ratio and exhibits weak binding to the β-adrenoreceptor, exerting a beta-blocking effect that enhances antitumor immunity and preserves NK cell function [28]. Patients receiving perioperative β-blockers have a lower rate of recurrence of postoperative cancer metastases.

It has been postulated that volatile anesthetics can suppress both adaptive and maladaptive immunity, especially of NK, while propofol has been reported to preserve host immunity. This notion has been supported by the recent literature reporting the potential benefit of propofol-based TIVA over volatile anesthesia on overall survival, but not on recurrence in operated cancer patients [29].

Propofol acts both on prostate tumor cells, inhibiting proliferation and promoting apoptosis, and at the immunological level, producing an anti-inflammatory effect and stimulating NK cell activity [30]. One of the mechanisms by which propofol exerts an antitumor effect on prostate cancer cells is through the inhibition of hypoxia-inducible factor 1α (HIF-1α). This transcription factor plays a fundamental role in cell adaptation to hypoxic environments, a frequent phenomenon at the tumor level due to uncontrolled and accelerated cell growth. There are several in vitro studies [31] that have shown that propofol produces a concentration-dependent inhibition of HIF-1α levels. As a result, it is associated with a decrease in the progression and development of metastases in prostate cancer. In addition, propofol suppresses the isoflurane-induced upregulation of hypoxia-inducible factor 1α,10 and inhibits tumor cell proliferation, migration and invasion [32].

Activation of androgen receptors is related to greater tumor progression and metastasis, with androgen inhibition being the treatment of choice in advanced prostate cancers. Propofol decreases androgen receptor activation [33] as well as decreases the occurrence of castration-resistant prostate cancer [34]. Isoflurane also decreases androgen receptor activation but stimulates HIF-1α activation.

There are in vitro studies that attribute to propofol a role as an adjuvant with the different chemotherapeutic agents used in prostate cancer. Qian et al. [33] demonstrated that propofol produces a decrease in resistance to docetaxel induced by hypoxia or isoflurane [34] as well as a decrease in the epithelial–mesenchymal transition (EMT). In line with this, Yang et al. [35] showed that propofol produces an inhibition of lncRNA HOTAIR that is related to a greater sensitivity to the effects of paclitaxel (Table 1).

At the clinical level, there is only one prospective study that compared TIVA with halogenated drugs (sevoflurane and isoflurane) that found no statistically significant differences in biochemical recurrences (45.2% inhaled versus 39.1% intravenous) [19].

### 2.2. Opioids

Since opioids are the standard treatment for pain, they are an important mainstay in anesthesia. However, there is a trend to limit the use of opioids in the perioperative period due to their numerous adverse effects, including nausea and vomiting, hyperalgesia, addiction and death, and to consider a multimodal approach to pain.

At present, there is concern about the protumor effects that opioids may have [36]. From an oncogenic point of view, opioid receptors have been associated with phenomena such as angiogenesis and neovascularization through various pathways [37]. In addition, underlying mechanisms involving Src-mediated phosphorylation and transactivation of the vascular endothelial growth factor receptor, as well as MAPK/ERK activation, resulting in an immunosuppressive effect, have been demonstrated [38].

Lennon et al. observed that synthetic opioids stimulate the endothelium, thus facilitating cell migration and proliferation, in addition to promoting angiogenesis with an increase in the release of VEGF [39]. The intraoperative administration of fentanyl and its relationship with metastatic recurrence is controversial. It has been suggested that fentanyl suppresses NK cell-mediated cytotoxicity, however conclusive evidence has not yet been published.

The mU opioid receptor (MOR) has been associated with the evolution of cancer. In fact, MOR overexpression is related to a higher incidence of cancer metastasis [40].

Multiple studies have linked cancer recurrence with the use of opioids in the perioperative period. However, the current evidence is less conclusive in the case of prostate cancer.

There are studies that have found less biochemical recurrence in patients undergoing radical prostatectomy by epidural anesthesia versus opioid-based anesthesia [41]. The studies by Biki et al. [42] and Scavonetto et al. [43] demonstrated a detrimental effect of opioids on metastatic recurrence and overall survival in patients with prostate cancer. Tsui et al. found significant differences when evaluating biochemical recurrence after radical prostatectomy depending on the use or not of opioids [44]. Moreover, Kampa et al. demonstrated in vitro that opioids were capable of inhibiting the growth of different cell lines of prostate cancer cells [45] (Table 1).

On the other hand, a recent clinical trial compared biochemical recurrence in 146 patients with prostate cancer scheduled for prostatectomy in two randomized groups: non-opioid anesthesia and opioid-based anesthesia. It was concluded that opioids do not modify biochemical recurrence rates or disease-free time in patients with an intermediate or high D’Amico risk after prostatectomy [18]. Neither did the work by Roiss et al. find significant differences in biochemical recurrence, metastasis or overall survival in patients who underwent prostatectomy with or without opioids [46]. Finally, the work of Wuethrich et al. found no differences in biochemical recurrence and overall survival despite finding less tumor progression in patients with analgesia through an epidural catheter [47].

### 2.3. Regional Anesthesia

The use of locoregional techniques for multimodal anesthesia and analgesia seems to increase the survival rates of cancer patients due to a decrease in the systemic response to surgical stress, since it is known that sympathetic activation has an immunosuppressive role that favors the development of metastases, as well as the sparing role of opioids and other anesthetic drugs such as halogenated ones [48]. Furthermore, local anesthetics alone can induce apoptosis in prostate cancer cells [49]. There are few studies that have compared regional anesthesia (with or without general anesthesia) with exclusive general anesthesia in prostate cancer treatment, being mostly retrospective.

A recent literature review concluded that in prostate cancer surgery, the risk of mortality is reduced with the use of regional anesthesia, but no association between the anesthetic technique and survival free of progression or biochemical recurrence was found [50]. The meta-analysis by Lee et al. [51] did not find statistically significant differences in biochemical recurrence, although it did in overall survival, favoring the regional anesthesia group in contrast with Wuethrich et al. [52], who found that regional anesthesia did not increase biochemical recurrence-free survival, cancer-specific survival or overall survival.

A study by Sessler et al. investigated whether the use of general anesthesia versus regional anesthesia and analgesia in cancer surgery can produce a reduced rate of recurrence or metastasis, due to its lower impact on the immune system. They examined opioid-sparing anesthesia (such as regional anesthesia) and its impact on recurrence rate compared to general anesthesia alone. They demonstrated that locoregional technique can attenuate the cytokine changes caused by surgical stress by producing an increase in the serum levels of IL-12 and IFN-γ and a decrease in IL-6 and IL-10. Therefore, the highest levels of IFN-γ/IL-12, important cytokines for the CMI response, are maintained during the perioperative period in favour of a Th1 environment [53] (Table 1).

The use of locoregional techniques for anesthesia and multimodal analgesia seems to increase the survival rates of cancer patients due to a decrease in the systemic response to surgical stress, since it is known that sympathetic activation plays an immunosuppressive role that favors the development of metastases from a distance, as well as for the sparing role of opioids and other anesthetic drugs such as halogenated ones [40].

## 3. Statins

Although statins are not part of the anesthetic arsenal, due to their importance and high prevalence in patients with prostate cancer, we considered its inclusion in this article. Years ago, the relationship between the accumulation of cholesterol in prostate tissues and prostate cancer was demonstrated [54]. Specifically, the deposit of cholesterol occurs in the cell membrane in lipid rafts, which could activate signaling pathways related to the development of this type of cancer [55] and decrease the expression of the PTEN tumor suppressor gene, activating the pathway of PI3K-AKT-mTOR, related to high-grade prostate cancer [56].

Statins reduce cholesterol by inhibiting 3-hydroxy-3-methyl-gluratil coenzyme A (HMG-CoA) reductase, a key enzyme in the mevalonate pathway and currently one of the most widely used drugs against this tumor. Deregulation of this pathway may increase the risk of prostatic tumor progression [57]. On the other hand, by lowering cholesterol levels, statins reduce lipid rafts, affecting the epidermal growth factor receptor, the androgen receptor and the AKT and JAK-SATA three pathways, thus suppressing the growth of tumor cells [58]. In addition, statins have been studied in relation to other cancers (breast [59], colorectal [60] and prostate [61]) due to their anticancer properties (decreased inflammatory response, antiangiogenic properties and induction of apoptosis) [62] (Table 1).

Murtola et al. did not demonstrate an association between prostate cancer risk and statins in their 2007 study [63] but found that the active use of statins improved survival compared with no use. The use of statins after cancer diagnosis was associated with a lower risk of death in a subsequent study [64]. Yu et al. reported that postdiagnosis use was associated with a decreased chance of death and was more marked in men who took statins before prostate cancer diagnosis [65].

Regarding the risk of recurrence after radical prostatectomy, Allot et al. found that statin treatment decreased the risk of biochemical recurrence by 36% [66]. On the contrary, Jeong et al. did not demonstrate an association between the use of atorvastatin in patients with high-risk disease after prostatectomy and those who took placebo [67]. Raval et al. in a meta-analysis stated that statins reduce biochemical recurrence in patients undergoing radiotherapy, but not in patients with radical surgery [68]. More recent studies have shown a 40% risk reduction in biochemical recurrence with the use of statins and better oncological outcomes after radical prostatectomy [69]. In two 2022 meta-analyses, Yin et al. were only able to show some benefit in biochemical recurrence-free survival in patients with high-risk prostate cancer but found no improvement in patients who were started on statins after curative treatment [70]. Sun et al. demonstrated that statin users were significantly less likely to experience biochemical recurrence after primary treatment, especially after radiation therapy (Table 1). A high serum cholesterol level was significantly associated with biochemical recurrence in this type of cancer [71].

## 4. Discussion: Towards a New Paradigm

Despite advances in hormonal treatments, most prostate cancer patients undergo surgery for radical prostatectomy [72]. During surgical manipulation, tumor cells will reach the bloodstream and become disseminated tumor cells (DTCs) with metastatic potential [4]. The metastatic potential of DTCs is determined by the TME [73]. The TME influences prostate cancer survival/progression by enabling the immune evasion of tumor cells mainly through the activation of the PD-1/PD-L1 axis [10].

In prostate cancer, mechanisms of tumor immune escape also include the suppression/exhaustion of tumor-infiltrating cytotoxic T lymphocytes, inhibition of suppressor NK cells and upregulation of immunosuppressive immune cells (regulatory T, M2 macrophage, myeloid-derived suppressor, dendritic, stromal and adipocytic cells).

IFN-γ (the most investigated factor), TGF-β, TNF-α, IL-6, IL-17, IL-15, IL-27, complement factor C5a and other soluble molecules secreted by TME components, as well as hypoxia, also favour metastatic development [74].

Experimental studies have revealed that the intracellular pathways ERK/MEK, Akt-mTOR, NF-kB, WNT and JAK/STAT are involved in the regulation of PD-L1 in prostate cancer. Blocking PD-1/PD-L1 signaling using drugs can prevent immune escape from the tumor [74].

Because the inflammatory and immunological status of the patient is critical for cancer recurrence, it is critical that surgeons and anesthesiologists understand these factors and apply them to perioperative management. For years there has been a growing interest in how drugs used in the perioperative setting may affect the modulation of SMT and impact the survival of the cancer patient undergoing surgery.

Current evidence supports the antitumor effect of total intravenous anesthesia (TIVA) with propofol in cancer surgery mainly due to the positive regulation of NK cell and T helper cell activity. In contrast, the use of volatile anesthetics appears to induce cancer cell proliferation and migration in in vitro studies. On the other hand, a recent meta-analysis reviewing the effect of perioperative regional anesthesia on cancer recurrence concluded that regional anesthesia did not reduce the rate of cancer recurrence in cancer surgery over general anesthesia in cancer patients [75]. Studies that have evaluated the effect of regional versus general anesthesia in patients operated on for prostate cancer have had mixed conclusions, being mostly negative for differences in overall survival, biochemical recurrence-free survival or disease-free time. Most of these studies are retrospective except for a secondary analysis in a prospective study [42,46,51,52,76,77]. Regarding opioid use, we found conflicting conclusions regarding disease-free time and overall survival [43,77].

This paper reviews the current evidence on the effect of anesthetic drugs in prostate cancer and although work on the effects of drugs used perioperatively as modulators of SMT appears promising, the current evidence does not support a change in anesthetic practice or the use of specific agents or techniques in order to reduce the risk of cancer recurrence in prostate cancer surgery. Overall, the impact of anesthetic technique on prostate cancer outcomes remains unclear [50]. This could be related to the fact that the studies performed have important limitations that make them difficult to interpret and most are retrospective, lack randomization and do not present standardized anesthetic protocols for the treatment groups. The most significant effects have only been found over long exposure times, much longer than most clinical anesthetic procedures. For most anesthetics, the effects are clearly related to the drug concentration and exposure time.

Thus, as yet, no anesthetic–analgesic technique has demonstrated a direct correlative causality between its use in the perioperative period and a reduction in prostate cancer recurrence or an increase in disease-free time. However, knowing the different signaling pathways through which these drugs can modulate phenomena such as cell migration, apoptosis and neovascularization, among others, can generate coadjuvancy with the rest of the therapeutic measures provided to the oncology patient.

Our research group proposes the need to interpret the use of anesthetics in relation to the prognosis of patients who are going to be operated on due to prostate cancer. It would also likely be beneficial to evaluate the use of anesthetics against other tumors as a non-curative therapeutic tool but coadjutant towards the reduction in tumor recurrence. At present, all works are focused not on knowing if different anesthetic drugs can help reduce cancer recurrence in patients, but if they reduce it per se equating the anesthetic action to surgical therapy or chemotherapeutic drugs. Although this may be the case in some types of tumors, being able to initially demonstrate a modulation that reduces the possibility of tumor recurrence should be enough to decide the ideal technique for each tumor disease, and in this case, for the choice of anesthetic drugs used for patients with prostate tumors. If we look at the example of cardioprotection by halogens, the modulation of different enzymatic pathways is enough to electively use inhaled drugs in this group of patients, although the complication of a coronary anastomosis is unavoidable.

Currently, progress in this field is based on new markers and mediators of high sensitivity and specificity, and with a fundamental weight in the process of tumor recurrence, which will allow us to evaluate in future studies if there really is an antitumor modulation that by itself does select a type of anesthetic technique/drugs, similar to cardiac surgery.

Further work is needed to identify biomarkers to better understand the influence of anesthetic technique on tumor progression and to select the optimal anesthetic drug for each cancer patient, generating an individualized anesthetic plan according to the characteristics of the patient and the tumor process. In this respect, the coding process may also be of great importance.

Our new paradigm on knowledge in this area is based on the proposed idea of anesthetic action as a weapon in helping reduce cancer recurrence, and we believe that through the use of non-coding RNA, we could be able to demonstrate it.

Non-coding RNAs, in particular microRNAs, play an increasingly important role in the fields of oncology and anesthesia. Current evidence suggests that the beneficial or detrimental effects of anesthetics during cancer surgery are mediated by genetic and molecular mechanisms [78]. Undoubtedly, bridging the gap between basic research findings and clinical data towards evidence-based treatment is challenging, but has enormous potential to improve outcomes for prostate cancer patients.

Review articles have already investigated the role of lncRNAs in prostate cancer and have proposed the use of lncRNAs as biomarkers [79]. LncRNAs are able to regulate the proliferation and metastasis of prostate cancer cells and are related to the regulation of the STAT3, NF-κB, PTEN, PI3K/Akt and miRNAs pathways [80,81].

Along these lines, it has been suggested that anesthetics may influence the surgical outcome of cancer through miRNA changes both positively and negatively [82,83]. The anesthetic effects on cancer cell biology, cancer immunity and cell-to-cell communication via miRNA in prostate cancer should be the next point of study.

## 5. Conclusions

Evidence is clarifying how deregulation of the tumor microenvironment can favour metastatic development. We know that in the perioperative period, the use of anesthetic/analgesic techniques plays an important role in the survival of cancer patients undergoing surgery by interacting with the immune system. We believe that the type of cancer should be considered as an important factor in the clinical decision to choose a specific anesthetic plan, which is why we need more studies that show pharmacological interaction with the prostate cancer cell, since most of the current works on this type of tumor are contradictory and do not have a high level of evidence. It is also necessary to carry out studies that analyze the influence of drugs such as dexmedetomidine, NSAIDs and beta-blockers on prostate cancer.

It would be essential to identify the relationships between the RNAs that favor the progression of prostate cancer and the modulations that the drugs used in the perioperative period produce on these RNAs; in this way, we would understand what effect the use of these anesthetics has on patients with prostate cancer and we could develop an antitumoral anesthetic plan.

## Figures and Tables

**Table 1:** Summary of drugs that may influence prostate cancer recurrence.

Drug	Effect on Cancer	Mechanism of Action	Pathway Described
Propofol	Anti-Inflammatory Effect	COX-2 Inhibition	COX Pathway
	Decreases the progression and development of metastases	Decreases HIF-1α expression (angiogenesis, glycolysis, proliferation)	HIF-1α
	Promotes apoptosis	MMP expression STAT 3	STAT 3
Halogenated Agents	Decrease androgen receptor activation	Decrease the occurrence of castration-resistant prostate cancer	HOTAIR
	Increase the expression of prometastatic and protumorigenic factors	Increase HIF-1α expression (angiogenesis, glycolysis, proliferation)	HIF-1α
	–	Increase HIF-2α expression	P13K/Akt/mTOR pathway
Opioids	Promote angiogenesis and neovascularization	Src-mediated phosphorylation VEGF	MAPK/ERK pathway activation
	Immunosuppressive effect	Suppress NK cell-mediated cytotoxicity	mU opioid receptor (MOR)
Regional Anesthesia	Decrease surgical stress and sympathetic activation	Attenuates the cytokine changes caused by surgical stress	Increases IL-12 and IFNγ Decreases IL-6 and IL-10
Statins	Decrease the expression of PTEN tumor suppressor gene	Upregulation of apoptotic pathways	PI3K-AKT-mTOR
	Suppress the growth of tumor cells	Decrease EGFR and androgen receptor	AKT pathways JAK-SATA 3

## References

[1] Cassinello F., Prieto I., del Olmo M., Rivas S., Strichartz G.R. (2017). Cáncer surgery: How may anesthesia influence outcome?. J. Clin. Anesth..

[2] Coffey J.C., Wang J.H., Smith M.J., Bouchier-Hayes D., Cotter T.G., Redmond H.P. (2003). Excisional surgery for cancer cure: Therapy at a cost. Lancet Oncol..

[3] Chang C.-Y., Wu M.-Y., Chien Y.-J., Su I.-M., Wang S.-C., Kao M.-C. (2021). Anesthesia and Long-term Oncological Outcomes: A Systematic Review and Meta-analysis. Anesth. Analg..

[4] Kinoshita T., Goto T. (2021). Links between Inflammation and Postoperative Cancer Recurrence. J. Clin. Med..

[5] Onuma A.E., Zhang H., Gil L., Huang H., Tsung A. (2020). Surgical Stress Promotes Tumor Progression: A Focus on the Impact of the Immune Response. J. Clin. Med..

[6] Zhang H., Kong Q., Wang J., Jiang Y., Hua H. (2020). Complex roles of cAMP–PKA–CREB signaling in cancer. Exp. Hematol. Oncol..

[7] Wall T., Sherwin A., Ma D., Buggy D. (2019). Influence of perioperative anaesthetic and analgesic interventions on oncological outcomes: A narrative review. Br. J. Anaesth..

[8] Murdoch C., Muthana M., Coffelt S.B., Lewis C.E. (2008). The role of myeloid cells in the promotion of tumour angiogenesis. Nat. Rev. Cancer.

[9] Chang S.-H., Liu C.H., Conway R., Han D.K., Nithipatikom K., Trifan O.C., Lane T.F., Hla T. (2003). Role of prostaglandin E_2_-dependent angiogenic switch in cyclooxygenase 2-induced breast cancer progression. Proc. Natl. Acad. Sci. USA.

[10] Choi H., Hwang W. (2022). Perioperative Inflammatory Response and Cancer Recurrence in Lung Cancer Surgery: A Narrative Review. Front. Surg..

[11] Müller-Edenborn B., Roth-Z’Graggen B., Bartnicka K., Borgeat A., Hoos A., Borsig L., Beck-Schimmer B. (2012). Volatile anesthetics reduce invasion of colorectal cancer cells through down-regulation of matrix metalloproteinase-9. Anesthesiology.

[12] Witjes J.A., Lebret T., Compérat E.M., Cowan N.C., De Santis M., Bruins H.M., Hernández V., Espinós E.L., Dunn J., Rouanne M. (2016). Guidelines on Muscle-invasive and Metastatic Bladder Cancer. Eur. Urol..

[13] Ploussard G., Shariat S.F., Dragomir A., Kluth L.A., Xylinas E., Masson-Lecomte A., Rieken M., Rink M., Matsumoto K., Kikuchi E. (2013). Conditional Survival After Radical Cystectomy for Bladder Cancer: Evidence for a Patient Changing Risk Profile over Time. Eur. Urol..

[14] Enlund M., Berglund A., Andreasson K., Cicek C., Enlund A., Bergkvist L. (2014). The choice of anaesthetic—Sevoflurane or propofol—And outcome from cancer surgery: A retrospective analysis. Upsala J. Med. Sci..

[15] Siegel R.L., Miller K.D., Wagle N.S., Jemal A. (2023). Cancer statistics, 2023. CA A Cancer J. Clin..

[16] D’Amico A.V., Whittington R., Malkowicz S.B., Schultz D., Blank K., Broderick G.A., Tomaszewski J.E., Renshaw A.A., Kaplan I., Beard C.J. (1998). Biochemical outcome after radical prostatectomy, external beam radiation therapy, or interstitial radiation therapy for clinically localized prostate cancer. JAMA.

[17] Zhang L., Wu B., Zha Z., Zhao H., Jiang Y., Yuan J. (2018). Positive surgical margin is associated with biochemical recurrence risk following radical prostatectomy: A meta-analysis from high-quality retrospective cohort studies. World J. Surg. Oncol..

[18] Rangel F.P., Auler J.O., Carmona M.J., Cordeiro M.D., Nahas W.C., Coelho R.F., Simões C.M. (2021). Opioids and premature biochemical recurrence of prostate cancer: A randomised prospective clinical trial. Br. J. Anaesth..

[19] Kim N.Y., Jang W.S., Choi Y.D., Hong J.H., Noh S., Yoo Y.C. (2020). Comparison of Biochemical Recurrence After Robot-assisted Laparoscopic Radical Prostatectomy with Volatile and Total Intravenous Anesthesia. Int. J. Med. Sci..

[20] Orriach J.L.G., Ponferrada A.R., Manso A.M., Imbroda B.H., Belmonte J.J.E., Aliaga M.R., Fernandez A.R., Crespo J.D., Perez A.M.S., Heredia A.F. (2020). Anesthesia in Combination with Propofol Increases Disease-Free Survival in Bladder Cancer Patients Who Undergo Radical Tumor Cystectomy as Compared to Inhalational Anesthetics and Opiate-Based Analgesia. Oncology.

[21] Choi W.-J., Baek S., Joo E.-Y., Yoon S.-H., Kim E., Hong B., Hwang J.-H., Kim Y.-K. (2017). Comparison of the effect of spinal anesthesia and general anesthesia on 5-year tumor recurrence rates after transurethral resection of bladder tumors. Oncotarget.

[22] Exadaktylos A.K., Buggy D.J., Moriarty D.C., Mascha E., Sessler D.I. (2006). Can Anesthetic Technique for Primary Breast Cancer Surgery Affect Recurrence or Metastasis?. Anesthesiology.

[23] Hasselager R.P., Hallas J., Gögenur I. (2020). Inhalation or total intravenous anaesthesia and recurrence after colorectal cancer surgery: A propensity score matched Danish registry-based study. Br. J. Anaesth..

[24] Benzonana L.L., Perry N.J., Watts H.R., Yang B., Perry I.A., Coombes C., Takata M., Ma D. (2013). Faculty Opinions recommendation of Isoflurane, a commonly used volatile anesthetic, enhances renal cancer growth and malignant potential via the hypoxia-inducible factor cellular signaling pathway in vitro. Anesthesiology.

[25] Reis S.T., Leite K.R.M., Piovesan L.F., Pontes-Junior J., Viana N.I., Abe D.K., Crippa A., Moura C.M., Adonias S.P., Srougi M. (2012). Increased expression of MMP-9 and IL-8 are correlated with poor prognosis of Bladder Cancer. BMC Urol..

[26] Chen X., Du Y., Lin X., Qian Y., Zhou T., Huang Z. (2016). CD4 + CD25 + regulatory T cells in tumor immunity. Int. Immunopharmacol..

[27] Hooijmans C.R., Geessink F.J., Ritskes-Hoitinga M., Scheffer G.J. (2015). A systematic reviewand meta-analysis of the ability of analgesic drugs to reduce metastasis in experimental cancer models. Pain.

[28] Zhang X., Li F., Zheng Y., Wang X., Wang K., Yu Y., Zhao H. (2019). Propofol Reduced Mammosphere Formation of Breast Cancer Stem Cells via PD-L1/Nanog In Vitro. Oxidative Med. Cell. Longev..

[29] Chang C.-Y., Chien Y.-J., Wu M.-Y., Kao M.-C., Wang S.-C. (2019). Comment on “Anesthetic technique and cancer outcomes: A meta-analysis of total intravenous versus volatile anesthesia”. Can. J. Anaesth..

[30] Jiang S., Liu Y., Huang L., Zhang F., Kang R. (2018). Effects of propofol on cancer development and chemotherapy: Potential mechanisms. Eur. J. Pharmacol..

[31] Tatsumi K., Hirotsu A., Daijo H., Matsuyama T., Terada N., Tanaka T. (2017). Effect of propofol on androgen receptor activity in prostate cancer cells. Eur. J. Pharmacol..

[32] Zhang W., Wang Y., Zhu Z., Zheng Y., Song B. (2018). Retracted: Propofol inhibits proliferation, migration and invasion of gastric cancer cells by up-regulating microRNA-195. Int. J. Biol. Macromol..

[33] Qian J., Shen S., Chen W., Chen N. (2018). Propofol Reversed Hypoxia-Induced Docetaxel Resistance in Prostate Cancer Cells by Preventing Epithelial–Mesenchymal Transition by Inhibiting Hypoxia-Inducible Factor 1*α*. BioMed Res. Int..

[34] Huang H., Benzonana L.L., Zhao H., Watts H.R., Perry N.J.S., Bevan C., Brown R., Ma D. (2014). Prostate cancer cell malignancy via modulation of HIF-1αpathway with isoflurane and propofol alone and in combination. Br. J. Cancer.

[35] Yang X., Qin J., Gong C., Yang J. (2021). Propofol enhanced the cell sensitivity to paclitaxel (PTX) in prostatic cancer (PC) through modulation of HOTAIR. Genes Genom..

[36] Edinoff A.N., Derise O.C., Sheppard A.J., Miriyala S., Virgen C.G., Kaye A.J., Niakan M., Cornett E.M., Kaye A.D. (2022). The Influence of Analgesic Modalities on Postoperative Cancer Recurrence. Anesthesiol. Pain Med..

[37] McDonald J., Lambert D. (2015). Opioid receptors. BJA Educ..

[38] Yamamizu K., Hamada Y., Narita M. (2015). κ Opioid receptor ligands regulate angiogenesis indevelopment and in tumours. Br. J. Pharmacol..

[39] Lennon F.E., Mirzapoiazova T., Mambetsariev B., Salgia R., Moss J., Singleton P.A. (2012). Overexpressión of the μ-opioid receptor in human non-small cell lung cancer promotes Akt and mTOR activation, tumor growth, and metastasis. J. Am. Soc. Anesthesiol..

[40] Singleton P., Mirzapoiazova T., Hasina R., Salgia R., Moss J. (2014). Increased μ-opioid receptor expression in metastatic lung cancer. Br. J. Anaesth..

[41] Lec P.M., Lenis A.T., Golla V., Brisbane W., Shuch B., Garraway I.P., Reiter R.E., Chamie K. (2020). The Role of Opioids and Their Receptors in Urological Malignancy: A Review. J. Urol..

[42] Biki B., Mascha E., Moriarty D.C., Fitzpatrick J.M., Sessler D.I., Buggy D. (2008). Anesthetic Technique for Radical Prostatectomy Surgery Affects Cancer Recurrence. Anesthesiology.

[43] Scavonetto F., Yeoh T.Y., Umbreit E.C., Weingarten T.N., Gettman M.T., Frank I., Boorjian S.A., Karnes R.J., Schroeder D.R., Rangel L.J. (2013). Association between neuraxial analgesia, cancer progression, and mortality after radical prostatectomy: A large, retrospective matched cohort study. Br. J. Anaesth..

[44] Tsui B.C.H., Rashiq S., Schopflocher D., Murtha A., Broemling S., Pillay J., Finucane B.T. (2009). Epidural anesthesia and cancer recurrence rates after radical prostatectomy. Can. J. Anaesth..

[45] Kampa M., Bakogeorgou E., Hatzoglou A., Damianaki A., Martin P.-M., Castanas E. (1997). Opioid alkaloids and casomorphin peptides decrease the proliferation of prostatic cancer cell lines (LNCaP, PC3 and DU145) through a partial interaction with opioid receptors. Eur. J. Pharmacol..

[46] Roiss M., Schiffmann J., Tennstedt P., Kessler T., Blanc I., Goetz A., Schlomm T., Graefen M., Reuter D.A. (2014). Oncological long-term outcome of 4772 patients with prostate cancer undergoing radical prostatectomy: Does the anaesthetic technique matter?. Eur. J. Surg. Oncol..

[47] Wuethrich P.Y., Thalmann G.N., Studer U.E., Burkhard F.C. (2013). Epidural Analgesia during Open Radical Prostatectomy Does Not Improve Long-Term Cancer-Related Outcome: A Retrospective Study in Patients with Advanced Prostate Cancer. PLoS ONE.

[48] Ehdaie B., Sjoberg D.D., Dalecki P.H., Scardino P.T., Eastham J.A., Amar D. (2014). Association of anesthesia technique for radical prostatectomy with biochemical recurrence: A retrospective cohort study. Can. J. Anaesth..

[49] Xuan W., Zhao H., Hankin J., Chen L., Yao S., Ma D. (2016). Local anesthetic bupivacaine induced ovarian and prostate cancer apoptotic cell death and underlying mechanisms in vitro. Sci. Rep..

[50] Lusty A.J., Hosier G.W., Koti M., Chenard S., Mizubuti G.B., Jaeger M., Siemens D.R. (2019). Anesthetic technique and oncological outcomes in urology: A clinical practice review. Urol. Oncol. Semin. Orig. Investig..

[51] Lee B.M., Ghotra V.S., Karam J.A., Hernandez M., Pratt G., Cata J.P. (2015). Regional anesthesia/analgesia and the risk of cancer recurrence and mortality after prostatectomy: A meta-analysis. Pain Manag..

[52] Wuethrich P.Y., Hsu Schmitz S.F., Kessler T.M., Thalmann G.N., Studer U.E., Stueber F., Burkhard F.C. (2010). Potential influence of the anesthetic technique used during open radical prostatectomy on prostate cancer-related outcome: A retrospective study. Anesthesiology.

[53] Sessler D.I., Pei L., Huang Y., Fleischmann E., Marhofer P., Kurz A., Mayers D.B., Meyer-Treschan T.A., Grady M., Tan E.Y. (2019). Recurrence of breast cancer after regional or general anaesthesia: A randomised controlled trial. Lancet.

[54] Schaffner C.P. (1981). Prostatic cholesterol metabolism: Regulation and alteration. Prog. Clin. Biol. Res..

[55] Hea Y.O., Eun J.L., Yoon S., Byung H.C., Kang S.C., Sung J.H. (2007). Cholesterol level of lipid raft microdomains regulates apoptotic cell death in prostate cancer cells through EGFR-mediated Akt and ERK signal transduction. Prostate.

[56] Yue S., Li J., Lee S.Y., Lee H.J., Shao T., Song B., Cheng L., Masterson T.A., Liu X., Ratliff T.L. (2014). Cholesteryl ester accumulation induced by PTEN loss and PI3K/AKT activation underlies human prostate cancer aggressiveness. Cell Metab..

[57] Clendening J.W., Pandyra A., Boutros P.C., El Ghamrasni S., Khosravi F., Trentin G.A., Martirosyan A., Hakem A., Hakem R., Jurisica I. (2010). Dysregulation of the mevalonate pathway promotes transformation. Proc. Natl. Acad. Sci. USA.

[58] Jung Y.Y., Ko J., Um J., Chinnathambi A., Alharbi S.A., Sethi G., Ahn K.S. (2021). LDL cholesterol promotes the proliferation of prostate and pancreatic cancer cells by activating the STAT3 pathway. J. Cell. Physiol..

[59] Ponferrada A.R., Orriach J.L.G., Ruiz J.C.M., Molina S.R., Luque A.G., Mañas J.C. (2021). Breast Cancer and Anaesthesia: Genetic Influence. Int. J. Mol. Sci..

[60] Poynter J.N., Gruber S.B., Higgins P.D., Almog R., Bonner J.D., Rennert H.S., Low M., Greenson J.K., Rennert G. (2005). Statins and the Risk of Colorectal Cancer. N. Engl. J. Med..

[61] Kumar A., Riviere P., Luterstein E., Nalawade V., Vitzthum L., Sarkar R.R., Bryant A.K., Einck J.P., Mundt A.J., Murphy J.D. (2020). Associations among statins, preventive care, and prostate cancer mortality. Prostate Cancer Prostatic Dis..

[62] Wong W.W.L., Dimitroulakos J., Minden M.D., Penn L.Z. (2002). HMG-CoA reductase inhibitors and the malignant cell: The statin family of drugs as triggers of tumor-specific apoptosis. Leukemia.

[63] Murtola T.J., Tammela T.L., Lahtela J., Auvinen A. (2007). Cholesterol-Lowering Drugs and Prostate Cancer Risk: A Population-based Case-Control Study. Cancer Epidemiol. Biomark. Prev..

[64] Murtola T.J. (2014). Statins and biochemical recurrence after radical prostatectomy—Who benefits?. BJU Int. Engl..

[65] Yu O., Eberg M., Benayoun S., Aprikian A., Batist G., Suissa S., Azoulay L. (2014). Use of Statins and the Risk of Death in Patients with Prostate Cancer. J. Clin. Oncol..

[66] Allott E.H., Howard L.E., Cooperberg M.R., Kane C.J., Aronson W.J., Terris M.K., Amling C.L., Freedland S.J. (2014). Postoperative statin use and risk of biochemical recurrence following radical prostatectomy: Results from the Shared Equal Access Regional Cancer Hospital (SEARCH) database. BJU Int..

[67] Jeong I.G., Lim B., Yun S.C., Lim J.H., Hong J.H., Kim C.S. (2021). Adjuvant low-dose statin use after radical prostatectomy: The PRO-STAT randomized clinical trial. Clin. Cancer Res..

[68] Raval A.D., Thakker D., Negi H., Vyas A., Salkini M.W. (2016). Association between statins and clinical outcomes among men with prostate cancer: A systematic review and meta-analysis. Prostate Cancer Prostatic Dis..

[69] Jarimba R., Lima J.P., Eliseu M., Carvalho J., Antunes H., da Silva E.T., Moreira P., Figueiredo A. (2020). Statins Prevent Biochemical Recurrence of Prostate Cancer After Radical Prostatectomy: A Single-center Retrospective Study with a Median Follow-up of 51.20 Months. Res. Rep. Urol..

[70] Yin P., Han S., Hu Q., Tong S. (2022). The association of statin use and biochemical recurrence after curative treatment for prostate cancer A systematic review and meta-analysis. Medicine.

[71] Sun J.-X., Liu C.-Q., Zhong X.-Y., Xu J.-Z., An Y., Xu M.-Y., Hu J., Zhang Z.-B., Xia Q.-D., Wang S.-G. (2022). Statin Use and the Risk of Prostate Cancer Biochemical Recurrence Following Definitive Therapy: A Systematic Review and Meta-Analysis of Cohort Studies. Front. Oncol..

[72] Sekhoacha M., Riet K., Motloung P., Gumenku L., Adegoke A., Mashele S. (2022). Prostate Cancer Review: Genetics, Diagnosis, Treatment Options, and Alternative Approaches. Molecules.

[73] Anderson N.M., Simon M.C. (2020). The tumor microenvironment. Curr. Biol..

[74] Palicelli A., Croci S., Bisagni A., Zanetti E., De Biase D., Melli B., Sanguedolce F., Ragazzi M., Zanelli M., Chaux A. (2021). What Do We Have to Know about PD-L1 Expression in Prostate Cancer? A Systematic Literature Review. Part 3: PD-L1, Intracellular Signaling Pathways and Tumor Microenvironment. Int. J. Mol. Sci..

[75] Lee Z.X., Ng K.T., Ang E., Wang C.Y., Shariffuddin I.I.B. (2020). Effect of perioperative regional anesthesia on cancer recurrence: A meta-analysis of randomized controlled trials. Int. J. Surg..

[76] Tseng K.S., Kulkarni S., Humphreys E.B., Carter H.B., Mostwin J.L., Partin A.W., Han M., Wu C.L. (2018). Spinal anesthesia does not impact prostate cancer recurrence in a cohort of men undergoing radical prostatectomy: An observational study. Reg. Anesth. Pain Med..

[77] Sprung J., Scavonetto F., Yeoh T.Y., Kramer J.M., Karnes R.J., Eisenach J.H., Weingarten T.N., Schroeder D.R., Eisenach J.H., Karnes R.J. (2019). Outcomes after radical prostatectomy for cancer: A comparison between general anesthesia and epidural anesthesia with fentanyl analgesia: A matched cohort study. Anesth. Analg..

[78] Filipów S., Laczmanski L. (2019). Blood Circulating miRNAs as Cancer Biomarkers for Diagnosis and Surgical Treatment Response. Front. Genet..

[79] Mirzaei S., Paskeh M.D.A., Okina E., Gholami M.H., Hushmandi K., Hashemi M., Kalu A., Zarrabi A., Nabavi N., Rabiee N. (2022). Molecular Landscape of LncRNAs in Prostate Cancer: A focus on pathways and therapeutic targets for intervention. J. Exp. Clin. Cancer Res..

[80] Yu Y., Gao F., He Q., Li G., Ding G. (2019). lncRNA UCA1 Functions as a ceRNA to Promote Prostate Cancer Progression via Sponging miR143. Mol. Ther. Nucleic Acids.

[81] Xing P., Wang Y., Zhang L., Ma C., Lu J. (2021). Knockdown of lncRNA MIR44352HG and ST8SIA1 expression inhibits the proliferation, invasion and migration of prostate cancer cells in vitro and in vivo by blocking the activation of the FAK/AKT/β-catenin signaling pathway. Int. J. Mol. Med..

[82] Ishikawa M., Iwasaki M., Sakamoto A., Ma D. (2021). Anesthetics may modulate cancer surgical outcome: A possible role of miRNAs regulation. BMC Anesthesiol..

[83] Perry N.J.S., Buggy D., Ma D. (2019). Can anesthesia influence Cancer outcomes after surgery?. JAMA Surg..

